# On the Viability and Potential Value of Stem Cells for Repair and Treatment of Central Neurotrauma: Overview and Speculations

**DOI:** 10.3389/fneur.2018.00602

**Published:** 2018-08-13

**Authors:** Samantha Wu, Kevin T. FitzGerald, James Giordano

**Affiliations:** ^1^Pellegrino Center for Clinical Bioethics, Georgetown University Medical Center, Washington, DC, United States; ^2^Department of Oncology, Georgetown University Medical Center, Washington, DC, United States; ^3^Departments of Neurology and Biochemistry, Georgetown University Medical Center, Washington, DC, United States

**Keywords:** nervous system trauma, neural stem cells, autologous transplantation, regeneration, cell and tissue based therapy, spinal cord injury, traumatic brain injury

## Abstract

Central neurotrauma, such as spinal cord injury or traumatic brain injury, can damage critical axonal pathways and neurons and lead to partial to complete loss of neural function that is difficult to address in the mature central nervous system. Improvement and innovation in the development, manufacture, and delivery of stem-cell based therapies, as well as the continued exploration of newer forms of stem cells, have allowed the professional and public spheres to resolve technical and ethical questions that previously hindered stem cell research for central nervous system injury. Recent *in vitro* and *in vivo* models have demonstrated the potential that reprogrammed autologous stem cells, in particular, have to restore functionality and induce regeneration—while potentially mitigating technical issues of immunogenicity, rejection, and ethical issues of embryonic derivation. These newer stem-cell based approaches are not, however, without concerns and problems of safety, efficacy, use and distribution. This review is an assessment of the current state of the science, the potential solutions that have been and are currently being explored, and the problems and questions that arise from what appears to be a promising way forward (i.e., autologous stem cell-based therapies)—for the purpose of advancing the research for much-needed therapeutic interventions for central neurotrauma.

## Neurotrauma: an overview

Neurotrauma is defined as neurological insult that results in the disturbance of neural circuitry through disruption of axonal pathways and/or neural cell damage or loss (1). Injuries to the central nervous system (CNS) include, but are not limited to, spinal cord injury (SCI), traumatic brain injury (TBI), and stroke (1–4).

The epidemiological impact of neurotrauma is significant. Annually, there are approximately 12,000 cases of SCI in the United States alone (5), with injury being incurred by compression, contusion, laceration and/or partial or complete severing of the cord (6). There are 1.5–2 million cases of traumatic brain injury (TBI) per year in the US, with 75,000–100,000 of these cases being classified as severe (7, 8). Approximately 795,000 strokes occur annually in the US, with 87% of these being ischemic and 10% due to intracranial hemorrhage (9). Neurotrauma can incur both short and long-term, partial or complete loss of neurological function, depending on the type and severity of injuries (7, 8, 10). Pathophysiological features can include sensory and/or autonomic impairment, muscle weakness, and/or decreased control of movement, decreased endurance, muscle spasms, and hypertonicity, and are typically reflective of the site(s) and extent of neurological damage (6–8, 11). Onset of signs and symptoms resulting from neurotrauma can begin immediately following injury, or in some cases (of mild to moderate insult, with iterative neurological involvement) can be latent, and can persist for months, years and/or durably (i.e., - permanently).

The short- and long-term effects of neurotrauma can extend beyond the biophysiological to detrimentally affect quality of life (i.e., employment opportunities, relationships, ability to partake in recreational activities, etc.) (12–14). Post-trauma care and management of symptoms can also be economically burdensome for individuals and families affected by SCI, TBI or stroke. For example, in the US, the first year of care following an SCI injury, can incur individual costs ranging from US$123,000 to US$423,000 (15). Lifetime costs for an individual injured at 25 years of age have been estimated to reach US$2.7 million, depending on the severity of the injury incurred (16).

While we recognize that SCI, TBI, and stroke each and all have differing therapeutic targets, and methods, and have been, and may be therapeutically approached in distinct ways, herein we take a heterogeneous approach to include all of these conditions in this review, as stem cells are and may be used in a variety of therapeutic applications. As well, we place specific emphasis on SCI, recognizing the particular potential for application of stem cell-based therapies in this context, and acknowledge and address that TBI and stroke have been shown to share certain pathophysiological processes and clinical targets, which have been supportive of stem-cell based interventions (3, 17, 18).

## The therapeutic/recuperative problem

Patterns of recovery of function following neurotrauma generally reflect neurons' incapacity for replication (i.e., post-mitotic stability). It is noteworthy that some areas of the brain have shown regenerative capacity (i.e., neurogenesis) (19–21) through initiation and migration of neural progenitor cells. Regions that have exhibited progenitor cell activation and resulting neurogenesis include the hippocampal subgranular zone (22–24) and dentate gyrus [(25); however, for contrasting report, see (26)], the periventricular area, the olfactory bulb (27–30), the subventricular zone (31–33), and the central canal of the spinal cord (34–37). However, progenitor cells appear to be isolated to these regions, and are not ubiquitous in the CNS. The majority of mature neurons of the CNS are terminally differentiated (TD), can no longer undergo mitosis, and are considered to be outside of the cell cycle (38). While there is some debate whether neurogenesis may persist into and throughout adulthood in certain brain areas [e.g., the dentate gyrus; (25, 26, 39)]; such mitotic capacity, even if limited to key periods in development, is certainly not neuroanatomically ubiquitous. In mammals (including humans) cortical neurons become mature, post-mitotic cells early on during development (40), and maintain the stable post-mitotic state for decades (41). Further evidence suggests that the human neocortex does not acquire any additional neurons after birth (41), and that brain tumors are not derived from mature, TD neurons, but rather, from aberrant neural stem cells found in the areas of the brain with regenerative ability (38, 42). Neuronal cell death also typically occurs when TD neurons are induced to re-enter the cell cycle, either experimentally or due to cell stress (38, 43).

Following neural insult and injury, the terminal zones of severed neurons swell into dystrophic growth cones and have been shown to have some regenerative capabilities when in the appropriate environment(s) (44–47). Neurons of the dorsal root ganglia, for instance, have axons in both the peripheral neural system (PNS) and the CNS; however, only those ending in the PNS have capacity for regeneration (48). The inability of CNS neurons to regenerate neural fibers is related to the environment of the adult CNS following injury (49). When a neuronal fiber is severed, axonal regrowth is initially inhibited by the presence of myelin-associated inhibitors in the glial environment [see (50, 51) for overview]. These myelin-associated inhibitors are released by both intact oligodendrocytes and myelin structures that may have been damaged by injury (47, 52–55). Identified inhibitors include: Nogo, myelin-associated glycoprotein (Mag), oligodendrocyte myelin glycoprotein (Omgp), ephrin B3 and transmembrane semaphoring 4D (Sema4D) (54, 56).

Following CNS injury, microglia, oligodendrocyte precursors, meningeal cells, and astrocytes are also recruited to the site. Activation of local inflammatory processes and induction of lipid-collagen matrices lead to gliosis and formation of a glial scar, which provides a physical barrier and further inhibits regrowth of the axon and outgrowth of neurites beyond the lesion site (1, 47, 54). Moreover, the recruited astrocytes enter a reactive state, in which they up-regulate and release chondroitin sulfate proteoglycans (CSPGs), creating a chemical gradient at the site of injury that is inhibitory to regrowth (47, 57, 58). Additional evidence suggests that the glial scar may also provide stability to the site of injury in the CNS, preventing further cellular degeneration, isolating the inflammatory response, and repairing the blood-brain barrier at the injury site (59–61).

Thus, the adult nervous system is largely unable to regenerate following irreparable neuronal damage and/or loss (1, 62–64). This presents challenges (viz. a “therapeutic/recuperative problem”) to developing effective interventions for neural insult, and is an increasing clinical (and socio-economic) concern as the incidence of neurological injury and global prevalence of neurodegenerative disease rise (1, 6, 38, 47, 54, 65).

## Cell-based approaches to central neural repair and regeneration

A variety of strategies have been developed to treat CNS injury and disease. In general, methods for the repair and possible regeneration of the CNS entail pharmacological, structural, and cell-based approaches, either singularly or in combination. Although pharmacological approaches have demonstrated therapeutic benefit for management of symptoms and functional rehabilitation associated with SCI (66), TBI (67), and stroke (68, 69), not all have been proven safe or effective in reparative or regenerative capacities in clinical application. Structural approaches, while perhaps limited in their reparative and regenerative capacities when employed in isolation, have demonstrated some success when employed in combination with cell-based approaches (70–74). We focus herein, however, on cell-based approaches to repair and possible regeneration of the injured CNS that have shown potential in preclinical and early clinical studies.

Cell-based therapies can have a variety (i.e., direct, indirect, or both types) of effects. Such therapies can be used to facilitate regeneration of neurons via implantation of specifically active cells in the adult CNS. Direct cell-based therapies generally involve use of implanted cells to replace or repair damaged neuronal and/or glial cells and tissue. Indirect effects involve use of implanted cells to contribute biomolecular and biochemical factors that modify the neural micro- and/or macroenvironment, providing trophic support that facilitates neural repair and regeneration (6, 75–77). Some cell-based therapies have both indirect and direct effects. Bone-marrow derived mesenchymal stem cells (BM-MSCs), for instance, exhibit immunosuppressive qualities and the ability to promote the proliferation of endogenous cells following stroke, in animal models (78–81). These cells are also capable of trans-differentiation into various neural cell types, such as astrocytes, oligodendrocytes, and neurons, both *in vitro* and *in vivo*, and in *in vivo* animal models have been shown to demonstrate migratory capacity and actions in the CNS (82–92).

### Stem cells

Stem cell-based therapies for neural regeneration and repair garnered attention after the identification of specific regions of the adult human brain capable of maintaining the capacity for neuroregeneration throughout the human adult lifespan (6, 77, 93–95).

Stem cell-based techniques have been increasingly innovative, with relatively rapid advances enabling the potential to combine stem-cell therapies with previously explored pharmacological, structural, and even other cell-based methods (96–99). For example, stem cells could be modified to deliver biomolecules or to replace damaged neurons, astrocytes, oligodendrocytes, etc. and thereby act directly and/or indirectly, as noted above (100).

As illustrated in Table 1, embryonic stem cells (ESCs), mesenchymal stem cells (MSCs), neural stem/progenitor cells (NSCs), and induced pluripotent stem cells (iPSCs) have all been explored for use in cell therapies for neuroregeneration in a variety of models and applications.

**Table 1:** Stem cell types (in addition to Schwann Cells and olfactory ensheating cells) being explored as treatment strategies for neuroregeneration and repair in neurotrauma (SCI, TBI, and stroke).

	**Source(s)**	**Potential progeny of differentiation**	**Benefits**	**Challenges**	**Specific examples of preclinical applications in neurotrauma (SCI, TBI, stroke)**	**Related references**
Embryonic Stem Cells and Fetal Stem Cells	Inner cell mass from blastocysts (donated from left over embryos from *in vitro* fertilization), therapeutic cloning/somatic cell nuclear transfer, or existing cell lines—currently 390 NIH-approved hESC cell lines and 70 unapproved; donated fetal brain tissue, umbilical cord blood, bone marrow; donated fetal brain tissue, umbilical cord blood, bone marrow	Pluripotent: Neural stem cells (NSCs), neural progenitor cells (NPCs), neurons and neuronal subtypes (dopaminergic, GABA, and motor neurons), glial subtypes (astrocytes, oligodendrocytes); note—some fetal stem cell sources demonstrate multipotency, with more limited differentiation profiles [i.e., neural progenitor cells, neurons, and neuronal subtypes (GABA neurons), glial subtypes (astrocytes)]	Pluripotent; almost indefinite proliferation *in vitro*; potential for *in vivo* migration, region-specific differentiation, and structural recovery following cell transplantation of ESCs and/or ESC-derived; some evidence of cognitive, motor, and sensory recovery in animal models of SCI, TBI, and stroke	Ethical: derivation of ESCs from leftover IVF embryos and therapeutic cloning/somatic cell nuclear transfer; limited supply; Medical: risk of undifferentiated cells and tumorigenicity; immune rejection; Technical: isolation and expansion of cells derived from fetal sources may be difficult; Financial: high cost	SCI: (101–115) TBI: (116–122) Stroke: (123–133)	(134–148)
Adult Neural Stem Cells	Post-mortem or adult brain tissue biopsy (subgranular zone of hippocampus; subventricular zone of striatum)	Multipotent: Neurons and neuronal subtypes (GABA neurons); glial subtypes (astrocytes) NG2-expressing NSCs can stimulate the generation of oligodendrocytes	Potential source of autologous cell transplants; proliferation *in vivo* and *in vitro*; cell replacement, improved motor functionality, neuroprotection and immunomodulation; neutrotrophic support	Technical: difficult isolation from biopsy/autopsy sample low numbers esp. of potent cells	SCI: (149–153) TBI: (154); Stroke: (155)	(31, 93, 94, 156–166)
Induced Pluripotent Stem Cells (iPSCs)	Skin fibroblasts, melanocytes, adipocytes, CD34+ cells, human primary keratinocytes, umbilical cord blood (CD133+cells) and peripheral blood mononuclear cells	Pluripotent: Neural stem cells, neural progenitor cells, neurons and neuronal subtypes (dopaminergic, GABA, and motor neurons), glial subtypes (astrocytes, oligodendrocytes)	Plentiful and multiple adult somatic cell sources; source of autologous NPCs; potential to differentiate and migrate *in vivo*; evidence of neuron replacement; axonal myelination; local trophic support; inhibition of further cell death; improved behavioral outcomes in mouse models	Technical: major manufacturing hurdles; uncertainty about epigenetic memory of iPSCs; optimization of reprogramming methods; screening and removal of undifferentiated cells prior to and after transplant; Medical: mutations, tumorigenicity, teratoma formation	SCI: (105, 114, 167–173); TBI: (171, 174–177); Stroke: (178–188)	(144, 189–223)
Schwann Cells	Sural nerve	Schwann cells that myelinate host nerves	Ability to isolate and culture Schwann cells from sural nerve; reduced risk of tumorigencity *in vivo*; axonal regeneration and functional recovery	Technical: optimizing grafted Schwann Cell—host astrocyte interactions to facilitate greater axon regrowth	SCI: (73, 224–230); TBI: (231, 232)	(233–235
Olfactory Ensheating Cells	Glial cells and NPC populations in the olfactory bulb	Olfactory ensheating glia; small numbers of NPCs inside the olfactory bulb	Migration from CNS to PNS; enhancement of axonal growth; potentially autologous source of cells; functional improvement	Medical: inconsistent results in clinical trials; Technical: need to establish a safe and effective injection/delivery method	SCI: (236–244) TBI: (245–247) Stroke: (248–250)	(74, 246, 251–253)

### Embryonic stem cells (ESCs), fetal stem cells and derivatives

ESCs were first cultured and isolated from mice (134), but can now be derived from donated human blastocysts following *in-vitro* fertilization (IVF) procedures (135, 136), somatic cell nuclear transfer (137), human or mice fetal brains (120, 122), or existing hESC lines (there are currently 390 NIH-approved hESC and 70 unapproved cell lines^1^

ESCs are pluripotent and can proliferate almost indefinitely *in vitro* (135, 138, 254). Furthermore, ESCs have potential to differentiate into any cell type, including neurotransmitter or growth factor-secreting cells, neural stem cells (NSCs) and neural progenitor cells that can be further differentiated into neuronal subtypes, and/or glia (e.g., oligodendrocytes, astrocytes) capable of effecting roles in facilitating neural repair and/or regeneration (117, 120, 121, 139, 254, 255).

Early preclinical studies employing *in vivo* mouse models demonstrated the ability of hESC-derived neural progenitor cells to integrate into host parenchyma, migrate along established pathways in the brain, and differentiate according to region-specific cues (254). Various cell transplantation applications of hESC-derived, as well as mouse or human fetal-derived NSCs, in animal models of TBI suggest the potential of these cells to migrate to injured regions of the brain, differentiate into neurons and neuronal subtypes, and improve cognitive and motor functional recovery in the injured brain (121, 122, 139). Transplanted ESC-derived cells in ischemic animal models (e.g., rats subject to middle cerebral artery occlusion (MCAO)) have also demonstrated the ability to differentiate *in vivo* and to improve structural, functional, behavioral, and motor and sensory repair (123–125). NSCs and NPCs derived from ESCs have also been applied in preclinical animal models of stroke (126–131) with marked improvements in the size of the infarct area, the level of differentiation into neurons and neuronal cell types post-transplantation, and improved behavioral deficits (256).

Transplanted ESC-derived NSCs have demonstrated functional and structural improvement in animal models of SCI, as well (101, 102, 104–107, 257, 258). An ongoing phase I/II study (NCT02302157) is investigating the application of human ESC-derived oligodendrocyte progenitor cells (OPCs) in subjects with subacute cervical SCI; this clinical trial is supported, in part, by preclinical evidence of the safety and ability of these cells to promote neurite outgrowth *in vitro* and to facilitate myelination *in vivo* in rodent models of thoracic SCI (103).

Risks of inappropriate differentiation (i.e., teratoma and tumor formation), as well as technical issues arising from the possibility of immunological and graft rejection of the implanted tissues are major challenges that are still to be overcome (140–142). As well, ethical controversies surrounding the source of ESCs has led to alternative sources of pluripotent stem cells being explored (143, 144).^2^

Fetally-derived NSCs can be isolated from fetal brain tissue, with minute quantities also reported in bone marrow and umbilical cord blood (259). While these cells readily give rise to neurons, astrocytes, and oligodendrocytes, they are less versatile and proliferative than ESCs because they maintain some of the characteristics of the region of the brain from which they were derived^3^ (100, 145). Transplantation of human fetal stem cells and their derivatives have shown promise in: increasing neuronal survival and decreasing lesion size in animal models of TBI through increased angiogenesis and reduced astrogliosis (122, 147); and, neuronal regrowth, functional and structural improvement following transplant in animal models of SCI (108–110). Clinical trials involving fetal stem cells or their derivatives for the treatment of SCI and stroke are completed and ongoing. A Phase I study is underway that is investigating the safety and efficacy of human fetal spinal cord-derived NSCs (NSI-566) for the treatment of chronic SCI (NCT01772810), and a Phase II study investigating the effect of human central nervous system stem cells (HuCNS-SCs) on patients with thoracic spinal cord injury has been completed (NCT01321333). Yet another Phase I/II clinical trial is ongoing that is evaluating the effect of NSI-566 cells on motor deficits in stroke patients (NCT03296618).

Despite such progress in preclinical and clinical fields, ESC- and fetally-derived stem cells have given rise to ethical concerns regarding the source and process of their procurement, technical considerations focal to possible risks incurred during transplantation, and unregulated tissue growth *in situ* [for overview, see (260)].

### A focus on neural stem cells and neural progenitor cells: possibilities and problems

NSCs are found in the developing and adult CNS in a number of brain regions [i.e., cortex, hippocampus, olfactory bulb, striatum, ventricles, midbrain, cerebellum, spinal cord, and retina, (31, 93, 94, 157–161). NSCs can be derived from ESCs, fetal tissues (as discussed in prior section), iPSCs, and adult brain tissue^1^ (160, 261). NSC differentiation (e.g., into neurons, astrocytes, and/or oligodendrocytes) is dependent, in part upon exposure to particular growth and environmental factors.

The use of adult NSCs/NPCs (derived from biopsy tissue from brain or spinal cord) in cell therapies could, if validated in preclinical proof-of-concept studies, allow for autologous cell transplantation and repair by endogenous NSCs, thereby potentially minimizing risks of incompatibility. Early studies in animal models of SCI and TBI have suggested the potential viability of this approach (isolation, *in vitro* expansion, and transplantation), either alone or in combination with other methods (e.g., bone marrow-derived MSCs) for provision of neuroprotection, immunomodulation and facilitation of re-myelination and functional recovery of the injured spinal cord (149–151, 153, 154, 156).

In transplantation-based therapy, NSCs or NPCs are implanted within defined areas of the CNS^1^. Transplanted NSCs and NPCs facilitate neuroregeneration and therapeutic plasticity in the injured CNS via processes of neuroprotection [i.e., the “bystander” “mechanism” in SCI, see (262) for overview; (132, 155) for ischemic stroke], neurotrophic support (262); immunomodulation (164), and cell replacement and integration (111). Broadly speaking, neuroprotection is the activation of pathways that prevent further neuronal cell death, while neurogenesis involves proliferation and differentiation of endogenous neural stem and progenitor cells (259, 262), or implanted NSCs capable of cell replacement. Lu et al. (113) found improved motor functionality following SCI in rodents, when rat- and human-derived NPCs, human ESC-derived NSCs, and human iPSC-derived NSCs were implanted at injury sites (112, 114, 115, 263). Specifically, it was observed that these cells were able to extend multiple axons over long distances, and form synapses with host neurons (112, 264). Research suggests that endogenous NSCs and the immune system share secreted mediators (chemokines, cytokines) and receptors relevant both to inflammation and the maintenance, structure, and function of endogenous neural stem cell niches in the brain (165, 166, 265). Transplanted exogenous NSCs may modulate local immune responses and secrete growth factors that are conducive to neuronal growth (132, 133). Indeed, both *in vitro* and *in vivo* proof-of-concept studies have demonstrated the potential of NSCs as a cell-based therapy for SCI (266–268), stroke (269), and TBI (266, 268, 269), based, at least in part, upon the capability for defined differentiation and target specific integration and functional activity of these cells when introduced at or proximate to sites of neural injury (259).

However, NSCs and NPCs from adult tissues cannot be easily engaged to generate certain types of neurons [e.g., dopamine or motor neurons; (162)]^2^, nor are they easily isolated or expanded from the regions of the human brain that contain NSCs [i.e., the subgranular zone of the hippocampus, and the subventricular zone of the striatum; (94)], in part, because they are present in rather low numbers (100). Moreover, despite the viability and potential value of transplanted NSCs as a therapeutic approach, gaps exist in knowledge about the activity and effect(s) of NSCs *in vivo* with regard to migration mechanisms and the optimum number of NSCs needed to facilitate regeneration in different types of lesions (166, 259). The method and timing of delivery, and potential interactions between the transplanted NSCs and the host immune system may also impact the induction and extent of neuroregenerative effects produced by these cells (160, 166).

The CNS environment at the site of neural injury in SCI, TBI, or stroke is not conducive to neuroregeneration (270). This is due to the activation of inhibitory pathways, glial scar formation, and the lack of guiding astrocytes essential for axonal regrowth (6). As a result, many applications of NSCs have resulted in poor cell survival, failed integration into host tissue, and poor, if not uncontrolled, differentiation of cells (6, 153, 271). Additionally, while NSC transplants derived from ESCs have shown increased risk for formation of tumor growth compared to NSCs that have been derived from either fetal or adult brain tissues, that is not to say that these cells do not also have associated risks of tumor formation^1^ (100, 260). Furthermore, given that a source of NSCs is from ESCs and fetal tissues, research and potential therapies using these stem cells generate concerns about derivation, cost, supply, and accessibility of these cells (11, 272, 273). In sum, these challenges, as well as mixed evidence of safety and efficacy in animal studies (152, 153, 274), have hindered translation of adult brain-derived NSCs/NPCs into clinical therapies (100, 259, 260).

### Translation of stem cell therapies

#### Ongoing trials

There are currently no stem cell therapies for neural injuries (namely, SCI, TBI, or stroke) that have received US FDA market approval for clinical application in humans, although there are 19 ongoing trials investigating stem cell therapies for SCI (as of May 2018; see Table 2 for details)^3^. Four of these are Phase 1, seven are Phase 1/2, six are Phase 2, one is phase 2/3, and one is unspecified. There are also 6 trials investigating stem cell therapies for TBI, and 22 trials investigating stem cell therapies for application to stroke^4^.

**Table 2:** Ongoing clinical trials as of May 2018 investigating stem cell therapies for SCI (Source:Clinicaltrials.gov).

**Title (ClinicalTrials.gov Identifier)**	**Responsible party/study location**	**Condition(s) of interest/intervention(s) of interest**	**Study overview**	**Phase, estimated completion, and status**
A Phase-1, Open-label, Single-Site, Safety Study of Human Spinal Cord-derived Neural Stem Cell Transplantation for the Treatment of Chronic SCI (NCT01772810) (275)	Joseph Ciacci (PI); UCSD Medical Center; Neuralstem Inc; San Diego, CA, USA	Spinal Cord Injury/human spinal cord stem cell implantation in paralysis patients with SCI	Seeking 8 participants; 4 to enroll in Group A (cord injury at T2-T12) or Group B (cord injury at C5-C7); 6 month study period post-op; follow-up period of 54 months	Phase 1/Recruiting; Estimated Completion: Dec 2022
The Efficacy and Safety of NeuroRegen Scaffold ™ Combined with Mesenchymal Stem Cells or Neural Stem Cells for Chronic Spinal Cord Injury Repair (NCT02688049) (276)	Jianwu Dai (PI); Chinese Academy of Sciences; Affiliated Hospital of Logistics University of CAPF, Tianjin, China	Spinal Cord Injury/NeuroRegen scaffold-MSC transplantation; Neuroregen scaffold-NSC transplantation	Randomized, double-blind clinical trial with 30 participants; two experimental arms (NeuroRegen scaffold-MSC transplantations OR NeuroRegen scaffold- NSC transplantation after SCI); in both arms, patients with chronic SCI (ASIA grade A) will receive NeuroRegen Scaffold with 10 million MSCs and NSCs, respectively; they will also undergo comprehensive rehabilitation,	Phase 2/Recruiting; Estimated Completion: June 2018
Safety and Efficacy of Autologous Neural Stem Cell Transplantation in Patients with Traumatic Spinal Cord Injury (NCT02326662) (277)	Alexander Averyanov (PI); Federal Research Clinical Center of Federal Medical & Biological Agency, Russia,	Spinal Cord Injury/Transplantation of autologous MSC-derived NSCs with a 3D biomatrix	Non-randomized interventional clinical trial involving 30 participants; testing the safety and efficacy of autologous MSCderived NSCs transplantation in paraplegic and tetraplegic patients in the acute, sub-chronic, and chronic phases of SCI;	Phase 1/2/Active, not recruiting; Estimated completion: Oct 2018
Transplantation of Purified Autologous Bone Marrow- or Leukapheresis-Derived CD34+ and CD133+ for Patients with Spinal Cord Injuries: A Long-Term Comparative Evaluation of Safety and Efficacy Study (NCT02687672) (278)	Stem Cells Arabia; Amman, Jordan	Spinal Cord Injury/Transplantation of unmanipulated, autologous CD34+ and CD133+ stem cells	Randomized interventional clinical trial seeking 50 participants for enrollment to one of two experimental arms (injection of leukapheresis-deprived, purified, autologous CD34+ and CD133+ stem cells OR injection of bone marrow derived, purified, autologous CD34+ and CD133+ stem cells); individuals will be monitored over a 60-month time period	Phase 1/2/Active, not recruiting; Estimated Completion: Dec 2021
The Effect of Intrathecal Transplantation of Umbilical Cord Mesenchymal Stem Cells in Patients with Late Stage of Chronic Spinal Cord Injury: A Multicenter, Prospective, Cohort Study (NCT03505034) (279)	Limin Rong; Third Affiliated Hospital, Sun Yat-Sen University, Guangzhou, Guangdong, China	Spinal Cord Injury/Intrathecal transplantation of Umbilical Cord MSCs	Part of a larger study to assess the safety and efficacy of intrathecal transplantation of allogeneic umbilical-cord derived MSCs for treatment of SCI in different phases; the present study will enroll 96 participants in a single group, assessing the intervention of interest in patients with late stage chronic SCI (more than 12 months post-injury); patients will receive	Phase 2/Recruiting; Estimated Completion: Dec 2018
A Phase II/III Clinical Trial to Evaluate the Safety and Efficacy of Bone Marrow-derived Mesenchymal Stem Cell Transplantation in Patients with Chronic Spinal Cord Injury (NCT01676441) (280)	Sangryong Jeon (PI); Pharmicell Co, Ltd.; Asan Medical Center, Songpagu, Seoul, Republic of Korea	Spinal Cord Injury/Autologous bone marrowderived mesenchymal stem cell transplantation directly into the injured spinal cord	Interventional single-center clinical trial with 32 participants; patients with cervical spinal cord injuries undergo a posterior cervical laminectomy and MSC transplantation; following laminectomy, 1.6 × (10^∧^7) and 3.2 × (10^∧^7) autologous MSCs are injected into the intramedullary and intrathecal space; subjects also receive 4 weeks of physical and	Phase 2/3; Active, not recruiting; Estimated Completion: Dec 2020
Umbilical Cord Mesenchymal Stem Cells Transplantation for the Treatment of Spinal Cord Injury (NCT02481440) (281)	Limin Rong; Third Affiliated Hospital, Sun Yat-Sen University, Guangzhou, Guangdong, China	Spinal Cord Injury/Subarachnoid injection of human allogeneic umbilical cord-derived MSCs	Randomized double-blind trial with 44 participants; two arms (experimental—patients with SCI receive intrathecal administration of up to 1 × (10^∧^6) umbilical cord MSCs per kg every month for 4 months; control—no umbilical cord MSC transplantation)	Phase 1/2; Recruiting; Estimated Completion: Dec 2018
The Effect of Intrathecal Transplantation of Umbilical Cord Mesenchymal Stem Cells in Patients with Sub-Acute Spinal Cord Injury: A Multicenter, Randomized, Controlled Trial (NCT03521336) (282)	Limin Rong; Third Affiliated Hospital, Sun Yat-Sen University, Guangzhou, Guangdong, China	Spinal Cord Injury; Intrathecal transplantation of umbilical cord-derived MSCs	Randomized trial with 130 subjects; single-masking; two arms (experimental—subjects receive intrathecal transplantation of umbilical cord MSCs (1 × (10^∧^6) cells/kg) once a month for 4 months; control—placebo sham operation with 10 ml saline, once a month for 4 months)	Phase 2/Recruiting; Estimated Completion: Dec 2022
The Effect of Intrathecal Transplantation of Umbilical Cord Mesenchymal Stem Cells in Patients With Early Stage of Chronic Spinal Cord Injury: A Multicenter, Randomized, Controlled Trial (NCT03521323) (283)	Limin Rong; Third Affiliated Hospital, Sun Yat-Sen University, Guangzhou, Guangdong, China	Spinal Cord Injury; Intrathecal transplantation of umbilical cord-derived MSCs	Randomized, single-masked, multi-center trial with 92 participants; two arms (experimental—subjects receive intrathecal transplantation of umbilical cord MSCs (1 × (10∧6)cells/kg) once a month for 4 months; control—placebo sham operation and 10 ml saline, once a month for 4 months)	Phase 2/Recruiting; Estimated Completion: Dec 2022
Randomized Clinical Trial for the Evaluation of Autologous Mesenchymal Stem Cells Transplantation in Thoracolumbar Chronic and Complete Spinal Cord Injury (NCT02574585) (284)	Ricardo Ribeiro-dos-Santos (PI); Hospital Sao Rafael, Brazil	Spinal Cord Injury/Autologous Mesenchymal Stem Cells Transplantation	Randomized, non-placebo controlled, prospective clinical trial enrolling 40 patients with SCI for at least 12 months with thoracolumbar chronic and complete spinal cord injury (ASIA grade A); two arms (experimental–20 subjects randomly assigned to receive 2 percutaneous injections of MSCs with a 3- month interval between injections; control−20 subjects randomly assigned to be clinically followed, without specific interventions)	Phase 2/Not Yet Recruiting; Estimated Completion: Jan 2022
Comparative Evaluation of Safety and Effectiveness of Autologous Bone Marrow Derived Mesenchymal Stem Cells (BM-MSC) vs. Adipose Tissue Derived Mesenchymal Stem Cells (AT-MSC) in the Treatment of Spinal Cord Injury (SCI) Patient (NCT02981576) (285)	Abdalla Awidi (Study Chair); Cell Therapy Center, University of Jordan, Amman, Jordan	Spinal Cord Injury/Autologous Mesenchymal Stem Cells Transplantation	14 SCI patients will be recruited and blindly divided into two different treatment groups (group 1: receive autologous MSCs from adipose tissue by intrathecal injection, 3x; group 2: receive autologous MSCs from bone marro by intrathecal injection, 3x)	Phase 1/2; Active, not recruiting; Estimated Completion: Jan 2019
Phase I Clinical Trial of Autologous Adipose Derived Mesenchymal Stem Cells in the Treatment of Paralysis Due to Traumatic Spinal Cord Injury (NCT03308565) (286)	Wenchun Qu (PI); Mayo Clinic, Rochester, MN, USA	Spinal Cord Injury/Paralysis/Autologous, adipose-derived MSCs (AD-MSCs)	Open-label, prospective safety and feasibility study of intrathecal injection of autologous culture-expanded adiposederived MSCs in patients with severe SCI; seeking 10 participants who will undergo a minor procedure to obtain adipose tissue for isolation and expansion of adipose-derived MSCs; autologous ADMSCs will then be transplated through intrathecal injection at L4-5 undero fluoroscopic guidance at single dose of 100 million cells; evaluation postinjection at day 2, 3, week 1, 2, 4, 24, 48, 72, and 96	Phase 1: Recruiting; Estimated Completion: Nov 2023
Evaluation of the Safety and Potential Effectiveness of Autologous Mesenchymal Stem Cells Transplantation in Subjects with Cervical Chronic and Complete Spinal Cord Injury (NCT02574572) (287)	Ricardo Ribeiro-dos-Santos (PI); Hospital Sao Rafael, Brazil	Spinal Cord Injury/Autologous Mesenchymal Stem Cells Transplantation	10 participants will be enrolled to a single group that will undergo laminectomy and autologous MSC intralesional injection	Phase 1; Recruiting; Estimated completion: June 2020
Open Label Study of Autologous Bone Marrow Mononuclear Cells in Spinal Cord Injury (NCT02009124) (288)	Alok K Sharma (PI); Neurogen Brain and Spine Institute, Mumbai, Maharashtra, India	Spinal Cord Injury/Autologous bone marrow mononuclear cell transplantation	Seeking to recruit 500 participants for a non-randomized, open label clinical trial with an experimental group (bone marrow is aspirated and mononuclear cells separated, injected intrathecally by standard lumbar puncture procedure) and control group (no stem cell therapy); will assess change in clinical symptoms and functional independence	Phase 2; Recruiting; Estimated completion: Dec 2018
Safety and Efficacy of NeuroRegen Scaffold™ with Bone Marrow Mononuclear Cells or Mesenchymal Stem Cells for Chronic Spinal Cord Injury (NCT02352077) (289)	Jianwu Dai (PI); Chinese Academy of Sciences; Affiliated Hospital of Logistics University of CAPF, Tianjin, China	Spinal Cord Injury/NeuroRegen scaffold with BMMCs or MSCs transplantation	30 participants, all patients with chronic SCI (ASIA grade A) receiving NeuroRegen Scaffold with BMMCs or MSCs transplantation, followed by comprehensive rehabilitation, psychological and nutritional measures; assessing improvements in neurophysiological measures, independence and quality of life, pain, urinary and bowel function	Phase 1; Enrolling by invitation; Estiamted completion: Dec 2018
A Phase I/Iia, Randomized, Double-blind, Single-dose, Placebo Controlled, Two-way Crossover Clinical Trial to Assess the Safety and to Obtain Efficacy Data in Intrathecal Administration of Expanded Wharton's Jelly Mesenchymal Stem Cells in Chronic Traumatic Spinal Cord Injury (NCT03003364) (290)	Joan Vidal (PI); Hospital de Neurorehabilitacio Institut Guttmann; Badalona, Barcelona, Spain	Spinal Chord Injury, Chronic/XCEL—UMCBETA (Expanded MSC from Wharton Jelly)	Phase I/Iia, randomized, double-blind, single-dose, placebo controlled, two-way crossover clinical trial; 10 patients (18–65 years old) affected by chronic traumatic SCI; patients in the experimental group will receive an intrathecal injection of XCEL-UMC-BETA, followed by placebo at month 6; patients in the placebo group will receive placebo until month 6, when they will receive XCEL-UMC-BETA	Phase 1/2; Active, not recruiting; Estimated completion: April 2020
Stem Cell Spinal Cord Injury Exoskeleton and Virtual Realtiy Treatment Study (NCT03225625) (291)	Jeffrey Weiss and Steven Silberfarb (Pis); Steven Levy (Study Director); The Healing Institute, Margate, Florida, USA	Spinal Cord Injuries/Bilateral paraspinal injects of BMSCs at the level of injury, superior and inferior to the spinal segment, followed by intravenous injection and intranasal placement; in addition to exoskeletal movement or virtual reality visualization	40 participants, non-randomized; assignedto one of three arms (Arm 1: BMSC paraspinal, IV, intranasal; Arm 2: BMSC paraspinal, IV, intranasal and exoskeleton; Arm 3: BMSC paraspinal, IV, intranasal and virtual reality); will assess autonomic nervous system function, quality of life, and ASIA Impairment Scale	N/A ; Recruiting; Estimated Completion: July 2022
Clinical Trial of Phase 1/2 to Evaluate the Feasibility, Safety, Tolerability and Preliminary Efficacy of the Administration of FAB117–HC, a Drug Whose Active Ingredient is HC016, Allogeneic Adipose Derived Adult Mesenchymal Stem Cells Expanded and Pulsed with H2O2, in Acute Traumatic SCI Patients (NCT02917291) (292)	Juana Maria Barrera Chacon (PI), Hospital Universitario Virgen del Rocio, Unidad de Lesionados Medulares, Sevilla, Spain; Miguel Angel Gonzalez Viejo (PI), Hospital Vall d'Hebron, Unidad de Lesionados Medulares, Barcelona, Spain; Antonio Rodriguez Sotillo (PI), Complexo Hospitalario Universitario A Coruna, Unidad de Lesionados Medulares, A Coruna, Spain	Acute Traumatic Spinal Cord Injury/FAB117– HC (HC016, allogeneic adipose-derived mesenchymal stem cells expanded and pusled with H2O2)	46 participants; randomized, double-blind study with 3 arms (FAB117–HC (ph1) – 3 patients who will receive an intramedullary administration of FAB117– HC (20 million cells) and 3 patients who will receive 40 million cells; Control group (ph2) of 20 patients receiving no treatment; FAB117-HC (Ph2) with 20 patients receiving intramedullary administration of the maximum tolerated dose (either 20 or 40 million cells)); assessing number of adverse events; changes in neurological function,	Phase 1/2; Recruiting; Estimated completion: Jan 2020
A Phase 1/2a Dose Escalation Study of AST-OPC1 in Subjects with Subacute Cervical Spinal Cord Injury (NCT02302157) (293)	Edward D Wirth III (Study Director); University of California at San Diego; Rancho Los Amigos/USC, Stanford University/Santa Clara Valley Medical Center; Shepard Center, Atlanta, GA; Rush University Medical Center, Chicago, IL; Indiana University, Indianapolis, IN; Washington University, Saint Louis, MI; Thomas Jefferson University/Magee Rehabilitation, Philadelphia, PA; Medical College of Wisconsin, Milwaukee, WI	Cervical Spinal Cord Injury, Spine Injury, Spinal Cord Trauma / AST-OPC1	Open label, single group assignment with 35 participants; participants will receive one injection of 2 or 10 million AST-OPC1 cells, or 2 injections of 10 million AST-OPC1 cells for a total of 20 million cells; cohort dependent; will assess number of adverse events within 1 year and neurological function	Phase 1/2; Active, not recruiting; Estimated Completion: Dec 2018

The majority (12) of the studies are investigating the safety and efficacy of MSCs for treatment of SCI, with the remainder utilizing NSCs [NCT01772810 (275) and NCT02326662 (277)], NSCs and MSCs (NCT02688049) (276), CD34^+^ and CD133+ stem cells (NCT02687672) (278), bone marrow mononuclear cells (BMMCs) (NCT02009124) (288), BMMCs and MSCs (NCT02352077) (289), and AST-OPC1 [human ESC-derived oligodendrocyte progenitor cells (OPCs)] (NCT02302157) (293). Seven (7) of these trials use allogeneic stem cells products [NCT03505034 (279), NCT02481440 (281), NCT02917291 (292), NCT03521336 (282), NCT03521323 (283), NCT03003364 (290), NCT02302157 (293)], 9 use autologous stem cells (NCT02326662 (277), NCT01676441 (280), NCT02687672 (278), NCT02574585 (284), NCT02981576 (285), NCT03308565 (286), NCT02574572 (287), NCT03225625 (291), NCT02009124) (288), and 3 are unspecified [NCT01772810 (275), NCT02688049 (276), NCT02352077 (289)]^4^.

Here we highlight several of these ongoing trials pertaining to stem cell therapies for SCI to demonstrate the types of studies being conducted. NCT02326662 (277) is a non-randomized interventional study assessing the safety and efficacy of MSC-derived autologous NSC transplantation for individuals with traumatic SCI (NCT02326662). The aim is to recruit 30 participants (paraplegic and tetraplegic patients in the acute, sub-chronic, and chronic phases of SCI), who will receive transplants of autologous MSC-derived NSCs by intraspinal and intrathecal injection, along with RMx Biomatrix scaffolding as needed. NCT02688049 is a Phase 1/2 randomized, double-blind clinical trial assessing the efficacy and safety of NeuroRegen Scaffold™ when combined with MSCs or NSCs for chronic SCI repair. It is a two-arm study, with patients in one arm receiving the NeuroRegen Scaffold™ combined with MSC transplantation therapy following SCI, and those in the other arm receiving NeuroRegen Scaffold™ combined with NSC transplantation following SCI (NCT02688049). NCT01676441 is a Phase 2/3 clinical trial being conducted in the Republic of Korea, that aims to evaluate the safety and efficacy of bone marrow-derived MSCs when transplanted into patients with chronic SCI. All participants (32) will receive MSC transplants directly to the injured spinal cord lesion site via laminectomy, and following recovery from the procedure, will undergo 4 weeks of physical and occupational therapy (NCT01676441). Participants will undergo magnetic resonance and diffusion tensor imaging and electromyography and nerve conduction testing at 6 months post-operatively, and be assessed for motor and sensory function as well as adverse events (using the American Spinal Injury Association (ASIA) scale) 12 months post-operatively (NCT01676441)^4^.

#### Reported results

At this writing, 13 trails involving stem cell therapies for SCI on ClinicalTrials.gov have been completed, although the results from all of these studies are not yet available. Park et al. (294) reported long-term outcomes (i.e., changes in motor power grade of extremities, MRI, and electrophysiological recordings) of 3 SCI patients who received intramedullary transplantations of autologous bone marrow-derived MSCs (sourced from the iliac bone of patients). The patients were identified from an initial cohort of 10 patients as those who showed evidence of improvement in their activities of daily living (ADL) at 6 months following MSC- transplant (295). Patients had received direct injections of MSCs (8 × 10^6^) to the spinal cord, in addition to introduction of MSCs (4 × 10^7^) to the intradural space. At 4 and 8 weeks post-operatively, patients received additional injections of MSCs (5 × 10^7^) via lumbar puncture. In this long-term follow-up, none of the 3 patients exhibited tumorigenesis or complications associated with the intramedullary injection of MSCs. Additionally, 2 of the 3 patients showed evidence of gradual improvement in motor power of the upper extremities, as well as evidence of the disappearance of cavity margins, which may suggest the ability of BM-derived MSCs to diminish glial scarring (294). The results of this study do not contradict those of previous studies [e.g., (296, 297); see (6) for an overview of completed trials involving stem cells for SCI], which similarly found slight motor improvements without significant complications associated with MSC transplantation in humans with SCI (294). The results of such studies suggest that stem cell therapies can be especially beneficial for functional recovery in the acute and subacute stages of SCI, but that improvements incurred during the later and chronic stages of SCI are characteristically less significant (6).

Clinical trials involving stem cell therapies for TBI and stroke have also been completed. Results for MSC transplants in TBI patients have been promising, with both (298) and (299) reporting enhancement of neurological function and recovery among adults and pediatric patients following injury. Kalladka et al. (300) recently reported on the use of human fetal brain-derived NSCs (CTX0E03) in patients with chronic ischemic stroke in a phase 1 study. This single-site, dose-escalation study found improved neurological function and no immunological or cell transplant-related adverse events in a sample of 13 men over the age of 60 who received intracerebral implants of (2, 5, 10, or 20 million) NSCs, up to 2 years post-transplant (300).

## More recent approaches: iPSCs and reprogrammed autologous cells

Alternatives to ESC and fetally-derived cell-based transplantation therapies received renewed attention upon the discovery that adult somatic cells could be induced to become pluripotent stem cells (190). Adult somatic cells include MSC derived from bone marrow, adipose, or epidermal tissues (301, 302). NSCs may also be induced from amniotic fluid stem cells (193). Induced pluripotent stem cells (iPSCs) have shown some promise in their ability to generate patient-specific, autologous pluripotent cells and tissues, although their ability to retain all autologous characteristics has recently come into question (194, 195). While iPSCs have been predominantly been used in animal and *in vitro* models, these studies have both significantly contributed to an understanding of neurodegenerative diseases, and are being viewed as significant resource tools for discovering novel therapeutic strategies against neurodegenerative disease and particular types of neurological injury (144, 303).

### iPSCs

Researchers successfully demonstrated that induced pluripotent stem cells (iPSCs) could be generated from mouse embryonic fibroblasts (190) and from adult fibroblasts (189, 191, 192, 199) that are cultured in the presence of some combination of four transcription factors (Oct4, Sox2, Klf2, and c-Myc; or Oct4, Sox2, Nanog, and Lin28)—rather than from embryonically- or fetally-derived cells (11, 144, 190, 191). Since then, iPSCs have become an attractive source of pluripotent stem cells for use in neural replacement and regeneration therapies, given their potential to generate a “virtually limitless supply” of autologous iPSCs—thus resolving ethical concerns regarding the source of stem cells and risks associated with immunological rejection in transplant recipients (11, 196). In neural therapies, iPSCs and their progeny can facilitate regeneration of neurons, induce axonal remyelination, provide trophic support and immunomodulation, and modify the extracellular microenvironment (105, 167, 177, 187).

### Reprogramming and differentiation of iPSCs for neural insult

The first step in generating iPSCs is to select an appropriate type of donor cell (201). Currently, the majority (i.e., 80%) of published studies of cellular reprogramming employ fibroblasts in that they are easy to obtain, purify, maintain, and have significant potential for autologous transplantation (11, 191, 273). Other somatic donor cells include: melanocytes, adipocytes, CD34+ cells, mesenchymal stem cells, human primary keratinocytes, umbilical cord blood CD133+ cells, and peripheral blood mononuclear cells (199, 208, 273, 304–307).

Each of these cell types has a characteristic differentiation profile related to its epigenetic memory (201, 308, 309). Such “memory” (e.g., particular patterns of DNA methylation, histone acetylation and phosphorylation) can influence patterns of expression and differentiation profiles of the resultant pluripotent stem cells (201, 309). Cells of different origins can also differ in their reprogramming efficiencies (11, 310). When designing cell therapies for neurotrauma, it is important to consider the donor cell source in light of the ease of isolation/expansion, the differentiation profile, the pathology of the CNS injury of interest, and the intended destination of the cell therapy in the injured patient.

Skin fibroblasts, for instance, are easily obtained but can require more time, resources, and manipulation to generate iPSCs due to their low programming efficiency (311, 312). Given the increased time required for reprogramming, they also have potential for increased mutations *in vitro*. Keratinocytes and melanocytes show therapeutic potential as sources of iPSCs: both are relatively easy to obtain from skin biopsies or plucked hairs, have relatively short reprogramming periods and high reprogramming efficiencies (203). Some research suggests the potential for these cells to be reprogrammed to neural stem cells, though further research is needed to improve the reprogramming process and validate its potential for differentiation and neuroregenerative applications (11, 313). Use of CD133+ cells from umbilical cord blood has been explored due to these cells ‘relatively high reprogramming efficiency and pluripotency (205); however, their use in therapies for CNS insult remains questionable, as there remains a need to characterize their NSC differentiation potential and optimal culture conditions (11, 314). CD34+ cells, derived from bone marrow and umbilical cord blood, are not recommended for clinical use due to their low availability and low reprogramming efficiency (206, 207). Stem cells derived from adipose tissue show notable promise as a reliable source of iPSCs: they are easily obtained during liposuction procedures, require fewer genomic changes to generate iPSCs, and the reprogramming period is both relatively short and efficient (208, 315).

Since the publication of studies by (189), a number of protocols for the development of iPSCs have become available that use these various adult-derived donor cells (some autologous) and employ different combinations of transcription factors (TFs) and delivery methods—with the goal of finding a protocol that optimally balances efficiency, safety and related risks (201, 311–321). Some TF delivery methods involve the use of viruses, such as lentiviruses, to integrate viral DNA into replicating regions of the host genome (322). The use of viral vectors to deliver reprogramming factors can, however, lead to chromosomal disruption, expression of transgenes, and/or mutations (322). More recent “scarless” technologies have been introduced that use episomal vectors, synthetic mRNAs and Sendai viruses that do not integrate to the genome during reprogramming of adult somatic cells into iPSCs (198, 323–325). Kim et al. (199) developed a vector-free method to generate human iPSCs directly from human fibroblasts through delivery of reprogramming proteins (Oct4, Sox2, Klf4, and c-Myc) fused to cell penetrating peptides capable of crossing the cell membrane (199). These “scarless” and vector-free systems reduce potential risks of chromosomal disruption associated with DNA transfection and genome manipulation.

Studies in animal models of SCI that involve transplantation of human NSCs derived from iPSCs also suggest that difficulties remain in ensuring *in vivo* differentiation of cells into appropriate neural cell lineages and with tumorigenicity—both of which may hinder successful cell engraftment and functional recovery (326, 327). Evidence suggests that the local CNS microenvironment and pro-inflammatory factors (and associated neuroinflammation) can influence transplanted cell engraftment, differentiation and survival (326). In light of this, optimization of protocols for human cell reprogramming and the generation of iPSCs for use in clinical trials remains a work in progress in an attempt to achieve balance in safety and efficiency when applied in humans for certain types of neurotrauma (11, 144, 328).

### Direct reprogramming and transdifferentiation of autologous somatic cells

Cell reprogramming studies published in the late twentieth and early twenty-first centuries that demonstrated transdifferentation challenged the widespread notion of predetermined cell fates (329–332). Indeed, NPCs/NSCs can be generated from iPSCs, but can also be directly generated from somatic cells (e.g., fibroblasts) via a process known as direct reprogramming (333, 334). Lujan et al. (335) showed the direct conversion of MEFs to NPCs using reprogramming factors (Sox2, FoxG1, and Brn2). Somatic cells can also be transdifferentiated to become mature neuronal cells [e.g., neurons, glia; (336, 337)]. Vierbuchen et al. (336), for instance, demonstrated that mouse embryonic fibroblasts (MEFs) could be directly converted to neurons. The same and other iterative combinations of factors (e.g., Musashi-1 (Msi-1)^5^) and techniques (e.g., silencing of donor cell-specific transcriptional programs, chromatin remodeling) to optimize cell transdifferentiation, generation and stability have been further identified (339–343).

Direct reprogramming via chromatin remodeling involves opening the chromatin in somatic cells, and facilitating sustained exposure to a set of transcription factors that effectively converts the somatic cells to NSC/NPCs; chromatin remodeling enables NSCs/NPCs to maintain their new identities and bypass the intermediate pluripotent state (337, 344, 345). For direct reprogramming approaches, bypassing the intermediate pluripotent state reduces the associated reprogramming risks of genetic and epigenetic abnormalities (346, 347). Continuing research is further exploring those combinations of TFs that could be most effective in direct reprogramming somatic cells into neuronal subtypes (e.g., dopaminergic, GABA-ergic, glutamatergic, and motor neurons), as well as distinct types of glial cells [i.e., oligodendrocytes, astrocytes, and Schwann cells; (325, 348)].

## Possible applications of autologous iPSC-derived cell types and drNPCs

NPCs have potential to participate in and facilitate central neural regeneration and repair by functioning to replace lost or damaged neurons, prompt axonal remyelination, and provide local trophic support (111, 132, 155, 262). Autologous iPSC-NPCs could be generated using a patient's somatic cells, and reprogrammed via dual SMAD inhibition (209, 349), embryoid body formation and differentiation into neural rosettes (210), or via the piggybac transposon system paired with induction of the NOTCH pathway (211, 324). Dimos et al. (350) generated autologous iPSCs from patient-specific fibroblasts that were then differentiated into motor neurons. As previously mentioned, drNPCs may also be generated via direct reprogramming of autologous somatic cells. In mouse models of SCI, transplanted iPSC-NPCs induced axonal remyelination and regeneration, inhibition of (further) cell death, and improvements in a number of behavioral outcomes (169, 170, 172). These cells have also shown the ability to differentiate *in vivo* into neurons and glial cells, migrate (169), and effectively integrate to host tissue within the CNS (114).

## Issues, questions, problems, and possible solutions

### Optimization of iPSC production and differentiation

Several important questions remain regarding reprogramming of human cells that must be considered pursuant to their use in clinical applications. For example, it will be important to discern to what extent iPSCs retain epigenetic memory of their donor cell types, and whether the induced pluripotency is equivalent to that seen in ESCs (144, 309, 351). It is also necessary to determine the extent to which different reprogramming methods (choice of donor cell and iPSC line, cell selection during propagation, and culture conditions) influences genetic variability and functionality of the differentiated cells (144, 310). While genetic variability between iPSC lines could theoretically be resolved via gene editing, human pluripotent stem cells have demonstrated resilience to conventional gene targeting methods, and even those methods that have worked (352–354) have been shown to be both inefficient and time consuming (144). Advancements made in the use of novel gene-editing systems, such as CRISPR/Cas-9 and its variants, may offer a possible solution to this demonstrated resilience in iPSCs to targeted gene-editing (144, 355).

### Mutations, tumorigenicity, and teratoma formation

Concerns regarding mutations and tumorigencitiy are focal to any consideration of the development and use of reprogrammed, differentiated iPSCs. iPSCs can acquire mutations during both the reprogramming process and *in vitro* expansion; furthermore, some protocols for generation of iPSCs call for the use of c-Myc—a proto-oncogene associated with various types of cancers (325, 356, 357). Risk of tumorigenicity and accumulation of genetic alterations might be reduced by selecting appropriate cell source(s) for the generation of iPSCs (i.e. adipose cells, keratinocytes, or melanocytes) (11, 310). Direct reprogramming may also offer a way to reduce these risks, as reprogrammed cells do not require an intermediate pluripotent stage (329–332).

Yet, even with sufficient controls for mutation during these phases of development, there remains a definitive need for an efficient technique for evaluating differentiation and screening for mutagenesis and potential tumor- and/or teratoma formation both prior to and following transplantation. Antibodies that target high-risk cell subpopulations and selective *in vivo* ablation of transplanted cells are potential solutions being explored to reduce this risk (358), and further studies are needed to more fully define the effectiveness of this, and other methods of insuring stability of these cells.

### Immunological interactions and possible rejection

A growing body of literature describes the numerous pathways of communication that exist between the immune system and the CNS—namely, the role that such communication has in the CNS' ability to maintain homeostasis and to react to neurological injury and trauma [e.g., neuroinflammation, (359–362)]. Communication between these two vital systems is mediated primarily through immune regulatory molecules that are released by cells residing in the CNS, such as microglia, mast cells, astrocytes, and oligodendrocytes (361–365).

Following neurotrauma, the CNS undergoes pathophysiological changes that both affect and are affected by the innate and adaptive arms of the immune system. After acute stroke, for instance, brain ischaemia, damage to tissue and subsequent release of pro-inflammatory factors (e.g., matrix metalloproteinase-3 (MMP-3), ATP, alpha-synuclein, and neuromelanin, (366, 367)) can activate innate immune (via leukocytes, toll-like receptors, and the lectin pathway) and neuroinflammatory responses, such as the release of cytokines and activation of resident microglial cells (359, 365, 368). T and B cells mediate the adaptive immune responses to neurological damage, providing both potentially protective and potentially damaging autoimmune/reactive effects on neural tissue, if inflammation is prolonged and unregulated [(362, 365, 369–371); for a thorough overview of the innate and adaptive immune responses to neurotrauma, see (359, 371)].

For cell therapies intended to treat the effects of neurotrauma, neuroinflammation and the potential for an immune response must be considered. In some cases, the administration of antibiotics, immunosuppresants, or other small-molecule anti-inflammatory agents [e.g., N-palmitoylethanolamine (PEA)] may be required, particularly following or alongside transplant or injection of tissue/cells into injured patients (362, 372, 373). However, one must also consider the effect that administration of such immunosuppressants may have on the stem cell therapies, cell differentiation and cell engraftment, as well as the optimum time for delivery of these substances (374).

In theory, the use of autologous adult somatic cells to generate iPSCs and progeny would avoid or at least significantly reduce immunogenicity in stem cell transplant therapies. However, Zhao et al. (375) suggested that transplantable stem cells could be subject to aberrant DNA methylation during the reprogramming process, and that such changes to the DNA might trigger an immune response after transplantation in the host. Other potential sources of immunogenicity may include culture reagents, protein expression, maturity of transplanted cells, or factors of the micro- or macro-environment at the site of transplantation (376). Others have explored the potential immunogenicity of autologous iPSCs using mouse (377, 378) and primate models (379), and these studies appear to contradict the results reported by Zhao et al. (375). Such ambiguity establishes the need to more stringently assess potential sources of immunogenicity in iPSC-derived cell therapies prior to their clinical use.

### Safety and use: addressing ethical, legal and social issues

Interest in stem cell research is sustained by the potential contribution that these cells and their progeny can make to transplant-based therapies for neurological repair and regeneration (260, 380, 381). Indeed, research suggests that stem cell transplantation affords potential as a therapeutic strategy for central neural injury and disease (260, 382, 383). Initially focused on use of ESCs as sources of pluripotent stem cells, the field was soon rife with ethical debate related to controversies surrounding the sources of ESCs, implications relevant to the status of early-stage human embryos (380), limited supply, high cost, and associated risks of tumorigenicity and immune rejection after transplantation.

In light of this, more recent basic and preclinical studies of regenerative therapies for CNS insult have shifted toward use of iPSCs and their derivatives that are generated from adult somatic cells. As noted above, iPSC-derived and directly reprogrammed neuronal and glial cell types can be generated from cell sources that are not (or at least less) controversial and more readily available. Such cell sources can be autologous—derived from tissue samples directly from patients—and thus, can eliminate or significantly reduce risks of immunological rejection after transplantation, and may appeal to current incentives for personalized and precision medicine. As well, developments have been made in reducing time spent *in vitro* during the reprogramming processes, improving selection of cell type, and increasing efficiency of methods to remove harmful cells pre- and post-transplantation, which, when taken together, can reduce risks of tumorigencity in iPSC transplant-based therapies.

To be sure, autologous reprogrammed cells have a number of attributes that make them attractive and potentially viable alternatives (to ESCs and fetal stem cells) in cell-based therapeutic approaches for particular types of CNS injury and disease. Yet, while this approach has potential to fulfill therapeutic goals of enabling partial to complete recovery of defined functions, ethico-legal and social issues persist. As described in detail elsewhere (260, 384, 385) such issues involve the unknowns of intermediate and long-term use of new techniques and technologies in practice. For example, (1) how such unknowns impact the validity and contingencies of informed consent; (2) the need for continuity of research and clinical care (to address emerging issues consequential to sustained use-in-practice); (3) legal liabilities and process of claims if/when adverse events occur; and (4) provision/distribution of resources and services (to furnish cells, as well clinical interventions, and ongoing support) in and across various populations in need.

We have proposed a risk assessment approach to more specifically define and address the issues, questions and problems generated by possible uses of emerging neuroscientific and neurotechnological developments (386, 387); but address does not assure resolution. While some concerns relate to safety and effectiveness (e.g., possibilities of unanticipated consequences, runaway effects) with regard for patients' welfare (i.e., via integrity of informed consent and availability of sustainable clinical care), others reflect recognition of problematic distribution of medical goods [for overview, see (388)]. Both safety and distribution can be met, to some extent, by rigorous quality control and expediency in the manufacture and delivery of cells. Still, it remains to be determined if the type and extent of broad-based medical care (i.e., trained personnel and institutional resources) can and/or will be made available to perform the procedures and insure sustained post-implantation evaluations and care.

We have opined, and re-iterate here, that any consideration of the ethics of biomedicine must regard economic factors, and in many cases, economics of biomedical research and care are determined by policy and law (389, 390). Here discussion of the viability and value of NSCs—or any biomedical technique and/or technology—centers upon the interactive roles of regulatory policy in establishing both standards of care and fiscal (i.e., insurance) subsidy of these approaches in patient care. Suffice it to note that the gravitas—and international relevance and importance—of this situation is such that a complete discussion of economic and policy issues focal to NSCs or translational neuroscience would be significant, and is therefore beyond the scope of this paper.

## Conclusions and future vistas

Research and possible use of stem cell therapies toward mitigating the effects of neurotrauma, and inducing neurological repair and regeneration are gaining momentum and attention—in both the professional and public spheres. Emerging techniques and technologies of cell development, manufacture and delivery are progressing at a considerable pace. A number of previously contentious technical and ethical issues and questions have been met and resolved, at least in part, by the advent of ever newer forms of stem cells. Of these, autologous stem cells appear to offer a number of advantages (e.g., immunological compatibility and lack of rejection, personalized therapy, non-fetal, or embryonic derivation) that would suggest their value in and for clinical use in effecting neurological repair and regeneration (following neurotrauma and in treatment of certain neurological diseases). Yet, it is likely that there will not be a single stem cell therapy for CNS injury, but instead a more customizable (pathology- and patient-specific) approach.

Regardless of iteration or approach, it will be important and necessary to develop and insure high quality, secure and rapid throughput manufacturing processes that enable time- and cost-efficient delivery of these cells. But manufacture is but one cog in a multi-spoked wheel of biomedical research and care. As noted, if developments in the brain sciences are to be translated into safe, affordable and accessible clinical care that maintain high quality, then medicine, as well as economics, regulatory policy and law must be aligned in order to support and sustain meaningful benefit to patients in need. Simply put, the prevalence and multi-dimensional burden of neurological injuries and neurodegenerative diseases demand the further exploration and pursuit of NSCs—and other therapeutic strategies—that are both promising and feasible.

## Author contributions

JG and KF developed the outline and structure of the work. SW conducted the literature review and drafted the initial manuscript. JG and KF oversaw subsequent revisions. JG, SW, and KF contributed and approved final edits and revisions to the manuscript for publication.

### Conflict of interest statement

The authors declare that the research was conducted in the absence of any commercial or financial relationships that could be construed as a potential conflict of interest.

## Footnotes

^1^National Institutes of Health, “NIH Human Embryonic Stem Cell Registry,” 4 May 2018, accessed May 2018, https://grants.nih.gov/stem_cells/registry/current.htm.).

^2^Mattis V, Svendsen S, Sareen D, and Svendsen C, “Neural stem cells,” Nature Neuroscience, 2010, https://www.stemcell.com/media/files/wallchart/WA10008-Neural_Stem_Cells.pdf.

^3^FDA, “FDA Warns About Stem Cell Claims,” 6 Jan 2012, accessed 8 Aug 2017, https://www.fda.gov/forconsumers/consumerupdates/ucm286155.htm#Regulation.

^4^ClinicalTrials.gov, accessed May 2018, www.clinicaltrials.gov.

^5^Musashi-1(Ms-1) is found to upregulate in injured ependymal cells in certain adult amphibians and lizards, and thought to play a role in regeneration of the injured spinal cord (143, 144, 338, 339).
